# Probiotic Bacillus Spores Together with Amino Acids and Immunoglobulins Exert Protective Effects on a Rat Model of Ulcerative Colitis

**DOI:** 10.3390/nu12123607

**Published:** 2020-11-24

**Authors:** Adrian Catinean, Maria Adriana Neag, Kiran Krishnan, Dana Maria Muntean, Corina Ioana Bocsan, Raluca Maria Pop, Andrei Otto Mitre, Carmen Stanca Melincovici, Anca Dana Buzoianu

**Affiliations:** 1 Department of Internal Medicine, Iuliu Hatieganu University of Medicine and Pharmacy, 400006 Cluj-Napoca, Romania; catinean@gmail.com; 2 Department of Pharmacology, Toxicology and Clinical Pharmacology, Iuliu Hatieganu University of Medicine and Pharmacy, 400337 Cluj-Napoca, Romania; corinabocsan@yahoo.com (C.I.B.); raluca.pop@umfcluj.ro (R.M.P.); abuzoianu@umfcluj.ro (A.D.B.); 3 Microbiome Labs, 101 E Town Pl, Saint Augustine, FL 92092, USA; kkiran_00@yahoo.com; 4 Department of Pharmaceutical Technology and Biopharmaceutics, Iuliu Hatieganu University of Medicine and Pharmacy, 400012 Cluj-Napoca, Romania; dana.muntean@umfcluj.ro; 5 Faculty of Medicine, Iuliu Hatieganu University of Medicine and Pharmacy, 400349 Cluj-Napoca, Romania; andrei.mitre97@gmail.com; 6 Histology Department, Iuliu Hatieganu University of Medicine and Pharmacy, 400349 Cluj-Napoca, Romania; carmen.melincovici@umfcluj.ro

**Keywords:** spore probiotic, *bacillus*, immunoglobulins, colitis, inflammation, amino acids

## Abstract

In inflammatory bowel disease (IBD), experimental models have proven to be important tools for evaluating potential therapeutic agents and for investigating the mechanisms of pathogenesis. Oxidative stress and the immune response have been associated with acetic acid (AA)-induced ulcerative colitis (UC). Our study aimed to evaluate, for the first time, the ability of a spore-based probiotic and an amino acid and immunoglobulin supplement in reducing tissue damage and inflammatory responses in an experimental animal model of UC. Forty-two Wistar rats were divided into six groups, receiving 1% carboxymethylcellulose, 4% AA, MegaSporeBiotic™ (MSB; 1 × 10^9^ colony forming units/day) and MegaMucosa™ (MM; 70 mg/100 g/day). Pretreatment with MSB or MM alone and in combination significantly lowered inflammation and reduced damage to the colonic mucosa. Pretreatment with these agents resulted in levels of proinflammatory cytokines, vascular tight junction proteins, and measures of oxidative stress similar to those reported for methylprednisolone, one of the first-line therapies for moderate to severe activity of UC. The protection was further confirmed by histologic analysis of the colon tissue. In conclusion, pretreatment with probiotic spore-forming *Bacillus* strains and a supplement of amino acids in combination with immunoglobulins exhibited anti-inflammatory and antioxidant effects in an AA-induced rat model of UC.

## 1. Introduction

Ulcerative colitis (UC) is a chronic inflammatory bowel disease (IBD), with an ever-increasing incidence and prevalence worldwide [1]. The disease poses particular concern due to an increased risk for patients with UC to develop colorectal cancer [2].

The aetiology of UC remains unclear, but multiple factors are thought to play a role. Environmental, genetic, and microbial factors have widely been acknowledged as likely being involved in the development of UC. Excessive inflammation and oxidative stress play a pivotal role in the pathogenesis of UC [3,4]. The pathophysiology of UC is characterized by the migration of neutrophils, basophils, and other leukocytes to both the mucosal membranes and superficial ulcers [5]. The release of inflammatory mediators such as cytokines and arachidonic acid metabolites, as well as free radicals, results in oxidative damage to the colonic tissue [6,7]. Patients with moderate or severe inflammation receive glucocorticoids (GC) as first-line therapy, which provides an important benefit in terms of both a reduction of disease activity and an induction of remission. GCs act on local bowel inflammation by reducing local cytokine and reactive oxygen species (ROS) production, thus limiting tissue damage [8]. However, this treatment often results in detrimental side effects, including severe and life-threatening infections [9].

The intestinal microbiome composition differs between patients with IBD and healthy subjects. For example, patients with IBD have a more predominant Proteobacteria population and decreased Firmicutes and Bacteroidetes populations [10,11]. Environmental factors may be involved in the development of microbiome dysbiosis; such dysbiosis may result in intestinal antigenic stimulus, causing chronic inflammation in genetically susceptible individuals [10,12]. The luminal microflora can be considered as a long neglected additional organ of the body; alterations in the microbial composition of the lumen have been implicated as driving elements of multiple intestinal and extraintestinal diseases [13,14]. In UC, the microbiota balance is altered when compared to healthy controls [12]. In the rise of the “microbiome era,” it is essential to elucidate the mechanisms of how immune and gut epithelial cells interact with and actively shape the intestinal microflora to maintain the gut immune homeostasis [15,16].

Probiotics are live microorganisms that help to re-establish and maintain microbial balance in case of dysbiosis. In particular, spore-based probiotics have proven to be more efficient than non-spore-based probiotics because of their increased resistance to stomach acids and because they can be stably stored at room temperature [17]. A spore-based probiotic formulation has been shown to reduce intestinal permeability, as measured via post-prandial endotoxemia, and the resulting inflammatory cytokines in healthy adults. These findings may illustrate a modulatory function in the microbiome by spore-based probiotics [18].

Another agent that has shown benefit to gut health is serum bovine immunoglobin (Ig)-containing protein (SBI) preparations. Oral administration of SBI preparations has been shown to promote weight gain, improve gut barrier function and permeability, and reduce the severity of enteropathy in animals [19]. SBI has been shown to influence the permeability of the intestinal barrier and to prevent antigen translocation by binding endotoxins and the microbial pathogens producing them [20]. The protective effect of SBI proteins on immune cell migration, cytokine production, and the host microbiota may be beneficial against chronic immune activation and persistent increased intestinal barrier permeability [21].

Also, amino acids are important nutrients both for maintaining the integrity of the intestinal mucosa and for supporting the growth of microorganisms in the intestine [22]. Besides, they have the ability to increase mucin production and stimulate mucin synthesis in the colon. This results in a thicker and healthier mucous barrier [23].

Taking these into account, we considered that these supplements would be a viable option for UC. Moreover, the effect of each supplement and the effect of their combination is important. Sometimes, both in the case of synthetic drugs and food supplements, positive or negative interactions can occur [24].

Our study aimed to evaluate, for the first time, the ability of a spore-based probiotic and a mucosal support supplement consisting of amino acids and Igs, either individually or in combination, to reduce both tissue damage and inflammatory responses in an experimental animal model of UC.

## 2. Materials and Methods

### 2.1. Agents and Chemicals

We used MegaSporeBiotic^TM^ (MSB) probiotic capsules (Microbiome Labs, Saint Augustine, FL, USA), MegaMucosa^TM^ (MM) powder (Microbiome Labs, Saint Augustine, FL, USA), and methylprednisolone (MP; 8 mg/tablet), which is a standard chemical compound. All products were purchased from a public pharmacy and administered orally as a suspension in 1 mL of 1% carboxymethylcellulose (CMC, vehicle). MSB is a probiotic blend of spores from five *Bacillus* species (*B. licheniformis, B. indicus, B. subtilis, B. clausii,* and *B. coagulans*) and MM is a combination of serum-derived Ig concentrate, amino acids (L-proline, L-serine, L-cysteine, L-threonine), and bioflavonoids. 

Analytical-grade acetic acid (AA) was purchased from Sigma-Aldrich (St. Louis, MO, USA) and tetraethoxypropane was purchased from ChemFaces (Wuhan, China).

### 2.2. Animals

Charles River Wistar albino male rats (*n* = 42) weighing between 230 and 270 g were obtained from the Center for Experimental Medicine and Practical Skills of Iuliu Hatieganu University of Medicine and Pharmacy. The rats were maintained under standard conditions of temperature (22 ± 2 °C), humidity, and light (12 h light/dark cycles). The animals were fed rat chow ad libitum and had free access to tap water. The rats were acclimated under these conditions for two days prior to starting the experiment. 

The working animal protocol was revised and approved by the Ethics Committee of Iuliu Hatieganu University of Medicine and Pharmacy (no. 26/25.02.2019) and by the National Sanitary Veterinary and Food Safety Authority (no. 160/11.03.2019). Specific regulations and amendments used in this study were from the “Guiding Principles in the Use of Animals in Toxicology” adopted by the Society of Toxicology (Reston, VA, USA) and all national laws regarding the protection of animals used for scientific research.

### 2.3. Experimental Design

**Figure 1:**
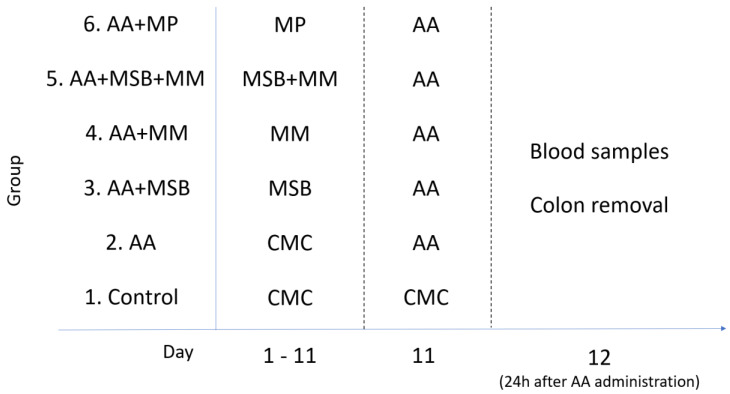
Experimental study design. Abbreviations: CMC, carboxymethylcellulose; AA, 4% acetic acid; MSB, MegaSporeBiotic™; MM, MegaMucosa™; MP, methylprednisolone.

A total of 42 Wistar rats were divided into 6 groups, with 7 rats per group. Group 1 served as the control and received the vehicle (1% CMC) only (11 days) and distilled water intrarectally; group 2 received 1% CMC for 11 days and 4% AA on day 11, serving as the model of colitis; group 3 received MSB (1 × 10^9^ colony forming units (CFU)/day, 11 days); group 4 received MM (70 mg/100 g/day, 11 days); group 5 received MSB (1 × 10^9^ CFU/day, 11 days) and MM (70 mg/100 g/day, 11 days); group 6 received MP (5 mg/kg/day, 11 days); additionally, groups 3, 4, 5, and 6 received 4% AA on day 11. All treatments except for 4% AA were administered orally through a feeding tube for 11 days; 4% AA was administered intrarectally. On day 12 (24 h after administration of 4% AA), blood and colons were collected for further analysis. Blood was collected from the retro-orbital sinus plexus (periorbital) under anesthesia. The blood was allowed to coagulate, then serum was separated by centrifugation (4000 rpm, 15 min, room temperature); the serum was stored at −20 °C for further biochemical analysis. Colon segments were preserved in 10% formaldehyde, dehydrated in graduated ethanol, and embedded into paraffin wax. Tissue blocks were sectioned at a 7 µm thickness and then deparaffinized, rehydrated (graded ethanol: 100%, 90%, and 80%), and stained with hematoxylin and eosin (H&E) for histologic analysis. The experimental design is shown in Figure 1.

### 2.4. Induction of Colitis

After an overnight fast, acute colonic inflammation (colitis) was induced in groups 2, 3, 4, 5, and 6 by intracolonic instillation of 4% AA. First, the rats were anesthetized with intraperitoneal ketamine (50 mg/kg). Next, 1.5 mL of 4% AA was administered (intracolonic) using a 6F silicone catheter tube connected to an injector that was advanced to 6–8 cm proximal to the anus verge. To prevent the solution from expelling, the animals were kept in a supine Trendelenburg position for 30 s.

### 2.5. Evaluation of Inflammatory and Cellular Markers

Serum levels of the proinflammatory cytokines interleukin-6 (IL-6) and tumor necrosis factor α (TNF-α) were quantified by enzyme-linked immunosorbent assay (ELISA) using commercially available ELISA kits (Rat TNF-α Standard TMB ELISA Development Kit, Rat IL-6 Standard ABTS ELISA Development Kit; PeproTech Inc., Rocky Hill, NJ, USA). The results were expressed as pg/mL. Serum levels of intercellular adhesion molecule 1 (ICAM-1), vascular cell adhesion molecule 1/CD106 (VCAM-1), and intestinal alkaline phosphatase (IAP) were also determined using commercially available rat-specific ELISA kits (Abbexa Ltd., Cambridge, UK). The results for ICAM-1 and IAP were expressed as ng/mL; VCAM-1 levels were expressed as pg/mL.

### 2.6. Assessment of Oxidative Stress

Oxidative stress may increase the vulnerability of the exposed tissues to injury. Plasma levels of malondialdehyde (MDA), a lipid peroxidation end-product [25], were determined to evaluate the level of oxidative stress. Plasmatic MDA bound to proteins was extracted by protein precipitation with 7% HClO_4_. After vortex-mixing and centrifugation (5 min at 10,000 rpm, 24 °C), the supernatant was transferred into autosampler vials and analyzed. Chromatographic separation was performed on a Zorbax C18 3 × 100 mm, 3.5 µm column that was maintained at 25 °C. The mobile phase consisted of 100% KH_2_PO_4_ 30 mM with the flow rate set at 0.5 mL/min. The absorbance of the eluent was monitored at 254 nm using HPLC system, Agilent 1100 series (Agilent Technologies, USA).

Total antioxidant capacity (TAC) levels were measured according to a validated method previously described by Erel [26]. The assay is based on the ability of antioxidants to decolorize the blue-green reagent, 2,2′-azinobis-(3-ethylbenzothiazoline-6-sulfonic acid radical cation) (ABTS^+^), according to their concentration and antioxidant capacities. The reduced ABTS molecule is oxidized to ABTS^+^ using hydrogen peroxide alone in acidic medium (acetate buffer: 30 mmol/L, pH 3.6). In acetate buffer solution, concentrated ABTS^+^ molecules (deep green) remain stable for a long period of time. Dilution with a more concentrated acetate buffer solution at a high pH (high pH acetate buffer: 0.4 mol/L, pH 5.8) results in a slow bleaching of the color. Antioxidants present in the sample accelerate the rate of bleaching in proportion to their concentration. This reaction can be monitored spectrophotometrically at 660 nm; the rate of bleaching is inversely related to the TAC of the sample. Trolox, a water-soluble analogue of vitamin E, was used to generate the calibration curve. This is widely used as a standard for TAC assays; results are expressed as mmol Trolox equivalent/L.

Catalase is an important enzyme that acts to dissociate hydrogen peroxide (H_2_O_2_) into molecular oxygen (O_2_) and water (H_2_O). Like other antioxidant enzymes, catalase is present in plant cells and animal cells such as erythrocytes, renal, and hepatic cells [27]. We used a UV-spectrophotometric method for measuring catalase activity. The principally common method for measuring catalase activity is the UV spectrophotometric method (Specord 250 Plus, Analytik Jena), which depends on monitoring the change of 240 nm absorbance at high levels of hydrogen peroxide solution (10 µmole/mL) dissolved in 50 mM phosphate buffer at pH = 7. High levels of hydrogen peroxide (H_2_O_2_) immediately lead to inhibition of the catalase enzyme by altering its active site structure. The method was previously described by Aebi [28].

### 2.7. Histological Assessment and Score

Histological evaluation of H&E-stained colon slices was performed using a Leica DM750 microscope (Leica Microsystems Ltd., Heerbrugg, Switzerland); images were captured using the Leica ICC 50 HD camera, which was connected to the microscope. Histological disease activity was assessed by evaluating both the inflammatory changes in the mucosa and mucosal architectural abnormalities.

We used the Robarts Histopathology Index (RHI) [29] to classify inflammation. The RHI assesses four main parameters: inflammation, presence of neutrophils in the lamina propria (≥1 neutrophils in the lamina propria), presence of neutrophils in the epithelium, and erosions or ulcerations. Each of these items is given a score from 0 to 3, then the total score is tallied, resulting in a range of 0–33 (1 × inflammatory infiltrate + 2 × lamina propria neutrophils + 3 × neutrophils in the epithelium + 5 × erosions or ulcerations). High scores may indicate severe inflammation.

A blind reading of histological sections was performed by two pathologists, independently. Four sections of each animal were evaluated and one from each animal used in the scoring. In our analysis, we evaluated the worst area of the colon tissue for each animal to calculate the RHI score [30].

### 2.8. Statistical Analyses

All data were presented as mean ± standard deviation (SD). The experimental design for all tested groups was done in such a way that first, an overall comparison between components was needed followed by comparisons between pairs of components. Thus, the differences between different groups was assessed using one-way analysis of variance (ANOVA) followed by post-hoc Bonferroni correction for multiple comparisons in order to identify the pairs that make the difference. To test the hierarchy between average values between groups, one tail t-tests were used. The level of significance alpha was set to 5%, so a *p*-value of less than 0.05 was considered statistically significant. All statistical analyses were performed using SPSS 10.0 statistical software (SPSS Inc., Chicago, IL, USA).

## 3. Results

### 3.1. TNF-α and IL-6 Levels 

**Figure 2:**
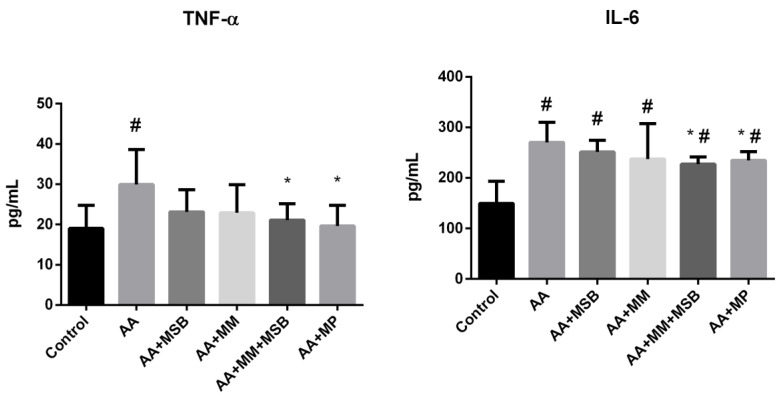
Serum TNF-α and IL-6 levels. * *p* < 0.05 compared to AA; # *p* < 0.05 compared to control. Abbreviations: TNF, tumor necrosis factor; IL, interleukin; AA, 4% acetic acid; MSB, MegaSporeBiotic^®^; MM, MegaMucosa^®^; MP, methylprednisolone. The bars represent mean values with standard deviations.

Serum TNF-α and IL-6 concentrations are shown in Figure 2. The serum concentration (mean ± SD) of TNF-α in healthy control rats was 19.00 ± 5.72 pg/mL. In the AA group (disease control), serum TNF-α levels were significantly higher compared with the healthy control group (29.92 ± 8.66 pg/mL; *p* = 0.009). In the AA + MSB and AA + MM groups, serum TNF-α levels were reduced by 22.6% (23.15 ± 5.44) and 23.4% (22.91 ± 6.92), respectively, relative to the AA group; these decreases were not statistically significant (*p* = 0.055 and *p* = 0.061, respectively). However, the combination of MSB and MM (AA + MSB + MM group) had a strong impact on TNF-α levels, which were significantly decreased (29.5%) relative to the AA group (21.10 ± 4.04; *p* = 0.018). The decrease in serum TNF-α observed for the AA + MSB + MM group relative to the AA group was similar to that observed for the AA + MP group (19.63 ± 5.09; *p* = 0.01).

The serum IL-6 concentration (mean ± SD) in the AA group was 270.58 ± 39.12 pg/mL, significantly higher than that of the healthy control group (149.64 ± 43.81 pg/mL; *p* < 0.001). Compared to the AA group, serum IL-6 was reduced in the AA + MSB and AA + MM groups (251.11 ± 23.16 and 237.51 ± 70.00 pg/mL, respectively), but the difference was not statistically significant (*p* = 0.141 and *p* = 0.151, respectively); the combination of MSB and MM (AA + MSB + MM group) resulted in a significantly lower (16.1%) serum IL-6 level (227.02 ± 14.50 pg/mL; *p* = 0.012), similar to that observed for the AA + MP group (13.16%) (234.96 ± 17.12 pg/mL; *p* = 0.029). 

### 3.2. ICAM-1, VCAM-1, and IAP Levels 

**Figure 3:**
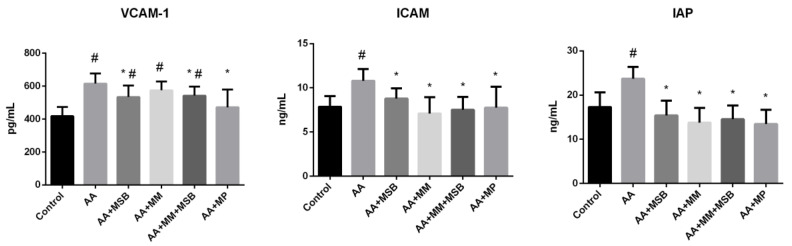
Serum VCAM-1, ICAM-1, and IAP levels. * *p* < 0.05 compared to AA; # *p* < 0.05 compared to control. Abbreviations: VCAM, vascular cell adhesion molecule; ICAM, intercellular adhesion molecule; IAP, intestinal alkaline phosphatase; AA, 4% acetic acid; MSB, MegaSporeBiotic™; MM, MegaMucosa™; MP, methylprednisolone. The bars represent mean values with standard deviations.

Serum VCAM-1, ICAM-1, and IAP concentrations are shown in Figure 3. The serum VCAM-1 concentration (mean ± SD) in the healthy control group was 417.48 ± 55.31 pg/mL. This level was significantly higher in the AA group (615.41 ± 61.40 pg/mL; *p* < 0.001). Serum levels of VCAM-1 were lower in all treated groups compared to the AA group, but the difference was only statistically significant in the AA + MSB, AA + MSB + MM, and AA + MP groups (*p* = 0.019, *p* = 0.018, and *p* = 0.006, respectively).

Compared to the healthy control group, serum ICAM-1 was significantly higher in the AA group (mean ± SD, 54.87 ± 1.22 versus 75.56 ± 1.32 ng/mL; *p* = 0.004). All treatment groups had a significantly lower level of serum ICAM-1 compared with the AA group.

Regarding IAP, mean ± SD serum levels were significantly higher in the AA group (23.72 ± 2.68 ng/mL) compared to the healthy control group (17.32 ± 3.32 ng/mL; *p* = 0.001). Compared to the AA group, serum IAP levels were significantly lower in all treated groups (*p* < 0.001 for all).

### 3.3. TAC, MDA, and Catalase 

**Figure 4:**
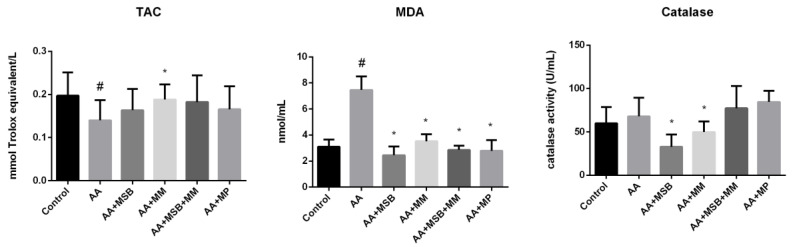
TAC, MDA, and catalase levels. * *p* < 0.05 compared to AA; # *p* < 0.05 compared to control. Abbreviations: TAC, total antioxidant capacity; MDA, malondialdehyde; AA, 4% acetic acid; MSB, MegaSporeBiotic™; MM, MegaMucosa™; MP, methylprednisolone. The bars represent mean values with standard deviations.

To explore the antioxidant capacity of the tested nutraceuticals (MSB and MM), TAC was measured. In the AA group, the TAC was significantly lower than the healthy control group (*p* = 0.02). As shown in Figure 4, a higher TAC was observed in all treated groups relative to the AA group; this difference only reached significance in the AA + MM group (*p* = 0.02). 

Plasma MDA (mean ± SD) was significantly higher in the AA group (7.46 ± 1.04 nmol/mL) compared to the healthy control group (3.09 ± 0.56 nmol/mL; *p* < 0.001). All study treatments had significantly lower plasma MDA levels relative to the AA group (*p* < 0.001 for all) (Figure 4).

Plasma catalase levels were significantly lower in the AA + MM and AA + MSB groups compared to the AA group (*p* < 0.05 for both), but slightly higher in AA + MSB + MM and AA + MP groups (Figure 4).

### 3.4. Histopathology

**Figure 5:**
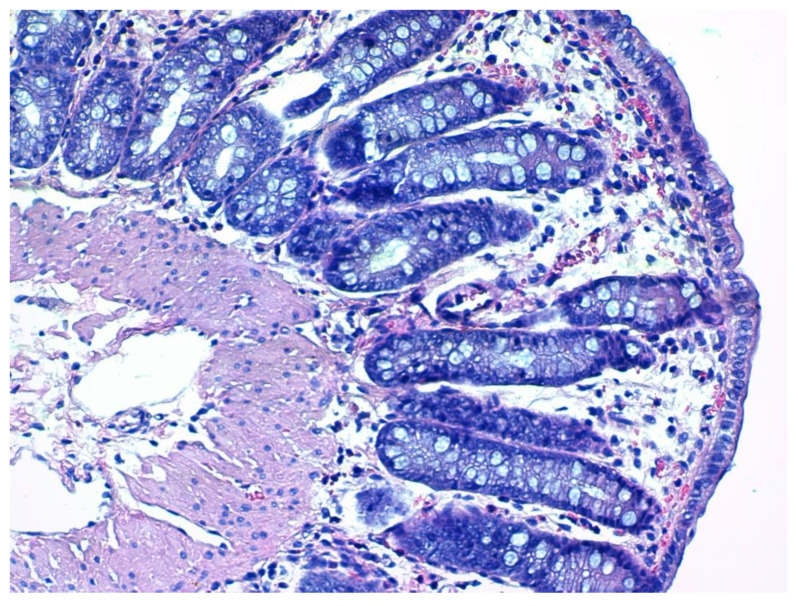
Colon histology of rats in the control group. Control group displayed a normal colonic mucosa, with intact and continuous surface epithelium and closely packed, thin, and long simple tubular colonic glands extending to the muscularis mucosa; small number of inflammatory cells in the mucosa and submucosa. The goblet cells are numerous in the colonic crypts. H&E stain, 20×. Abbreviations: H&E, hematoxylin and eosin.

**Figure 6:**
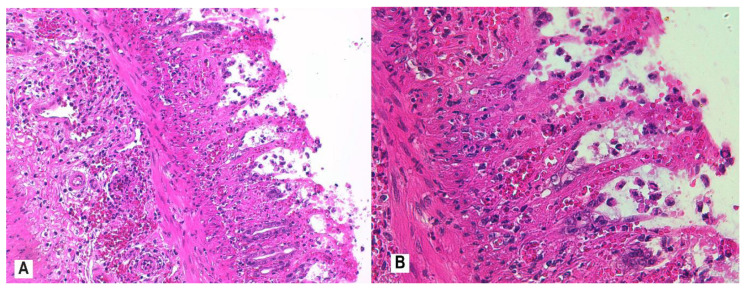
Colon histology of rats with active colitis (AA group). Colon tissue of rats 24 h after colonic exposure to AA demonstrates inflammatory infiltrates in the mucosa and submucosa, moderate levels of neutrophils in lamina propria, neutrophil infiltration of crypt epithelium in >50% of crypts, unequivocal erosion with loss of epithelial lining, and distortion of glandular architecture with goblet cell depletion; H&E stain, 20× (**A**), 40× (**B**). Abbreviations: AA, acetic acid; H&E, hematoxylin and eosin.

Control group histology is represented in Figure 5. Mucosal architectural abnormalities include distortion and shortening of intestinal crypts, and decreased crypt density with goblet cell depletion. Colon tissue slices from all rats in the AA group showed evidence of severe active inflammation (RBI > 20; Figure 6A,B).

**Figure 7:**
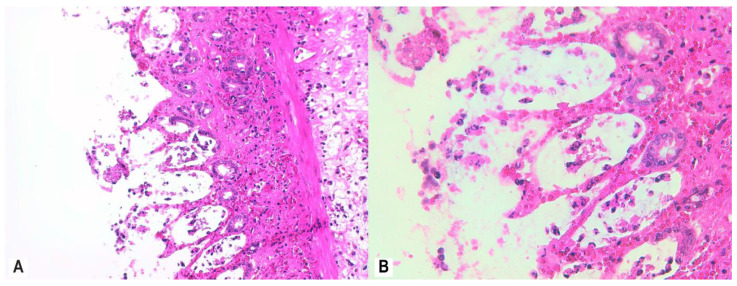
Colon histology of rats administered MSB (AA + MSB group). Colon tissue of rats that were administered MSB for 11 consecutive days prior to AA exposure was collected 24 h after AA exposure and demonstrated moderate inflammatory infiltrate in the mucosa and submucosa, few neutrophils in the lamina propria, neutrophil infiltration of crypt epithelium in <50% of crypts, unequivocal erosion with loss of epithelial lining, and distortion of glandular architecture with goblet cell depletion; H&E stain, 20× (**A**), 40× (**B**). Abbreviations: AA, acetic acid; H&E, hematoxylin and eosin; MSB, MegaSporeBiotic™.

**Figure 8:**
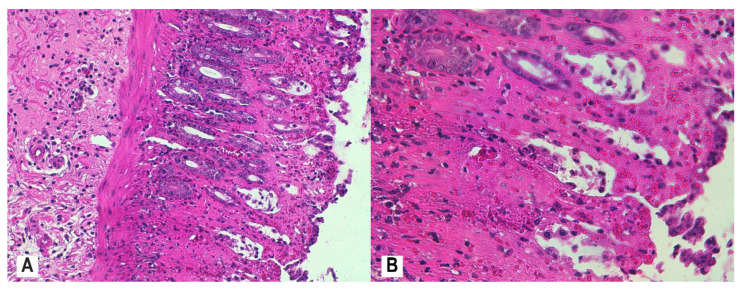
Colon histology of rats administered MM (AA + MM group). Colon tissue of rats that were administered MM for 11 consecutive days prior to AA exposure was collected 24 h after AA exposure and demonstrated moderate inflammatory infiltrate in the mucosa and submucosa, few neutrophils in the lamina propria, neutrophil infiltration of crypt epithelium in <50% of crypts, and recovering luminal epithelium; H&E stain, 20× (**A**), 40× (**B**). Abbreviations: AA, acetic acid; H&E, hematoxylin and eosin; MM, MegaMucosa™.

To assess mucosal damage, we evaluated the histological response according to RHI scores after treatment with MSB, MM, and MP. MSB resulted in a mild histologic improvement of the colonic mucosa (RBI < 20) (Figure 7A,B)*,* while MM administration showed a mild to moderate histological response (RBI < 16) (Figure 8A,B) relative to the AA group.

**Figure 9:**
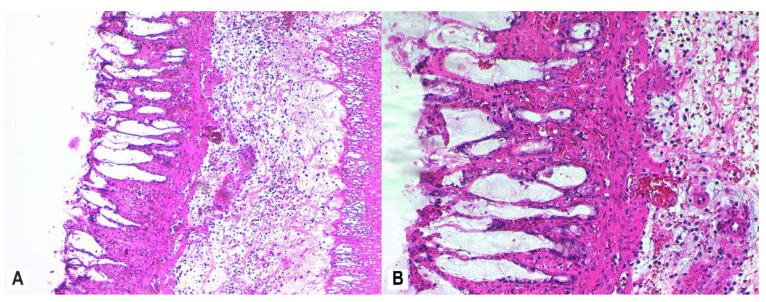
Colon histology of rats administered both MSB and MM (AA + MSB + MM group). Colon tissue of rats that were administered MSB and MM for 11 consecutive days prior to AA exposure was collected 24 h after AA exposure and demonstrated moderate inflammatory infiltrate in the mucosa and submucosa, absence of neutrophils in the lamina propria, neutrophil infiltration of crypt epithelium in <5% of crypts, recovering luminal epithelium, and increased crypt density and restoration of intestinal crypt architecture; H&E stain, 10× (**A**), 20× (**B**). Abbreviations: AA, acetic acid; H&E, hematoxylin and eosin; MSB, MegaSporeBiotic™; MM, MegaMucosa™.

**Figure 10:**
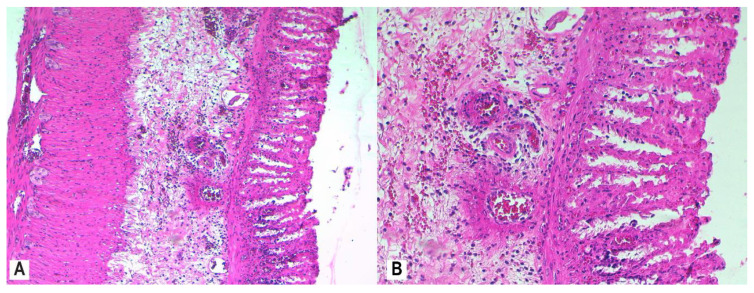
Colon histology of rats administered MP (AA + MP group). Colon tissue of rats that were administered MP for 11 consecutive days prior to AA exposure was collected 24 h after AA exposure and demonstrated moderate inflammatory infiltrate in the mucosa and submucosa, absence of neutrophils in the lamina propria, neutrophil infiltration of crypt epithelium in <5% of crypts, marked recovering luminal epithelium, and increased crypt density and restoration of intestinal crypt architecture; H&E stain, 10× (**A**), 20× (**B**). Abbreviations: AA, acetic acid; H&E, hematoxylin and eosin; MP, methylprednisolone.

A notable histologic protection of the colonic mucosa was achieved with combined MSB and MM administration (RBI ≤ 9) (Figure 9A,B) or with MP administration (RBI ≤ 9) (Figure 10A,B), with evidence on luminal epithelial changes, intestinal crypt architecture, and <5% of crypts having epithelial neutrophil infiltration. 

**Figure 11:**
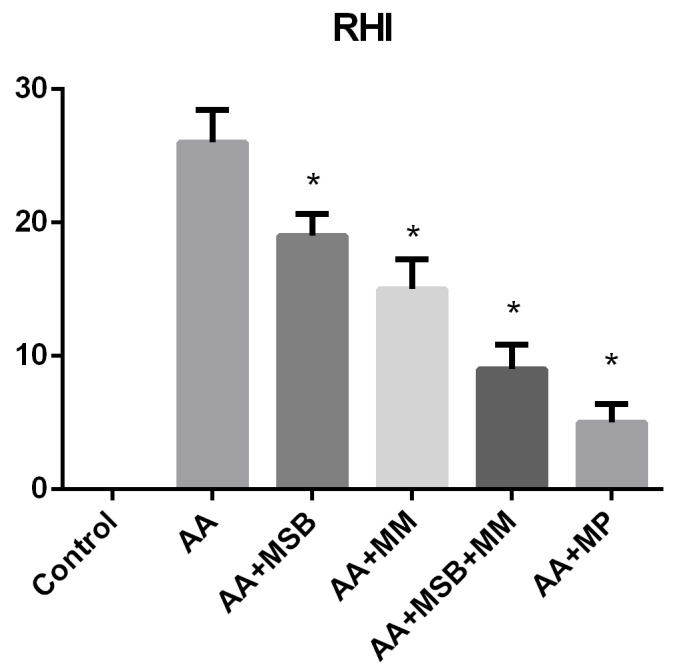
Robarts Histopathology Index. Robarts Histopathology Index was assessed by scoring for each animal the most affected area. Control samples were normal, with an index of 0, while AA samples had a mean score of 26. AA + MSB scored a mean of 19, AA + MM a mean of 15, AA + MSB + MM a mean of 9, and AA + MP a mean of 5. * *p* < 0.05 compared to AA. Abbreviations: RHI, Robarts Histopathology Index; AA, 4% acetic acid; MSB, MegaSporeBiotic™; MM, MegaMucosa™; MP, methylprednisolone. The bars represent mean values with standard deviations.

Histopathological Robarts indexes and their statistical analysis is represented in Figure 11.

## 4. Discussion

Spores have the ability to colonize the mucosa. Some spores, which have a heterologous antigen on their surface, have the ability to generate anti-tetanus toxin fragment C response. The immune response is only for those spores that have germinated and resporulated. This suggests that spores are not transient passengers of the gastrointestinal tract but rather have adapted to carry out their entire life cycle within this environment. Even if the number of *Bacillus* is lower compared to *Lactobacillus* or *Bifidobacteria* species, it should be clear that *Bacillus* species, for example *B. subtillis*, have an important role in the lymphoid tissue associated with the intestine. It has also been shown that specific sporulation with certain species of *Bacillus* (e.g., *B. clausi*) can influence the development of lymphoid tissue associated with the intestine [31].

We report the first study of the protective effect of MSB, MM, or a combination of the two against experimental UC. Acid-induced UC is a well-recognized model for the study of IBD that has been widely used to mimic the pathophysiological process of UC [32,33]. Because AA exposure initiates an inflammatory response by activating the cyclooxygenase and lipoxygenase pathways, we considered important to compare the effect of MSB and MM with MP, a well-known anti-inflammatory agent that acts through these pathways [34].

Decreasing the overexpression of inflammatory mediators in the intestine may benefit colitis [35]. Our study showed that the combination of MSB and MM resulted in a reduced level of the proinflammatory cytokines TNF-α and IL-6 compared to no treatment; the reduction was similar to that observed for MP (Figure 2). These results are in line with a study that evaluated the effect of spray-dried plasma and immunoglobulin (Ig) concentrate in a rat model of intestinal inflammation in which the dietary supplements prevented an increase of proinflammatory mediators in both the mucosa and serum of rats during inflammation [33,36].

The inflammatory process that occurs in UC is associated with altered intestinal barrier integrity and altered mucosal immune homeostasis. Additionally, UC has been associated with intestinal microbiome dysbiosis that may occur before the onset of the disease [37]. We speculate that MSB and MM are able to decrease inflammation by influencing intestinal permeability, which we showed in a previous study, where MSB decreased intestinal permeability and influenced the expression of zonula occludens (ZO-1) [38]. ZO-1 levels were positively correlated with proinflammatory cytokine levels (TNF-α, IL-1β) [38]. Duysburgh et al. used an in vitro simulation of the human gastrointestinal tract (M-SHIME^®^) to demonstrate that MSB influences the composition and activity of the gut microbiome, increasing the concentrations of short-chain fatty acids (SCFA; acetate, propionate, butyrate) in the colon and promoting the growth of *Bifidobacterium* spp., *Lactobacillus* spp., *Faecalibacterium prausnitzii*, and *Akkermansia muciniphila* [39]. Together, these findings could explain the results we obtained. On the one hand, MSB increases colonic butyrate [39], which helps maintain the structure of the intestinal barrier and blocks the abnormal expression of ZO-1, decreasing endotoxemia [38]. On the other hand, MSB influences the level of *F. prausnitzii*, a bacterium that is considered promising for the treatment of colitis [40], in an experimental model [39]. It has also been reported that serum-derived bovine Igs attenuated the effects of experimentally induced colitis (e.g., decreased number of goblet cells in the colon and reduction of mucin expression) in rats [41]. Our results showed a greater anti-inflammatory effect when MSB was co-administered with MM. This could be explained by the biological properties of the components of MM, as amino acids such as Lys and Thr are essential for intestinal microbes to achieve optimal growth [42].

MM and MSB, both individually and in combination, resulted in lower serum ICAM-1 levels and, to a lesser extent, lower serum VCAM-1 levels, both of which contribute to intestinal inflammation. The inflammation observed with IBD is promoted and sustained by the breakdown of the endothelial cell barrier in blood vessels, which results in the leaking of leukocytes from the blood into the intestinal tissue. Local micro vessels in the IBD mucosa are exposed to proinflammatory cytokines such as TNF-α and present an increased leukocytes adhesion. Platelets are important for endothelial function during inflammation. Endothelial cells can be activated through CD40 platelets, causing the upregulation of ICAM-1 and VCAM-1 [43,44]. Therefore, it is conceivable that there was a reduction of leukocyte adhesion in response to treatment with MSB, MM, or both, which was related to the downregulation of ICAM-1. In our model, assessments of mucosal inflammation were made 24 hours after the administration of AA, when the acute phase of the inflammatory response predominates; ICAM-1 plays an important role in this phase of inflammation. VCAM-1 is considered an important mediator, especially for chronic inflammation, when the recruitment of leukocytes predominates [45]. A possible explanation for the positive effect of MSB on ICAM-1 and VCAM-1 levels could be that MSB increases the concentration of butyrate [46], which, at physiological concentrations, inhibits the cytokine-induced expression of ICAM-1 and VCAM-1. It has also been reported that the cytokine-induced activity of NF-κB in endothelial cells is inhibited by butyrate [47]. Additionally, a study showed that NF-κB activation and IL-6 production were inhibited by cysteine in human coronary endothelial cells, exerting anti-inflammatory effects during endothelial inflammation [48].

Another important molecule that we evaluated in our study was IAP, which plays a major role in intestinal homeostasis and has the ability to inactivate (dephosphorylate) LPS both in the lumen and in blood [49]. Significantly lower serum IAP levels in the groups treated with MSB, MM, or both compared to those in the AA group may be caused by the prophylactic administration of these substances prior to the induction of colitis, which may preserve and even improve intestinal and systemic health, thus contributing to a reduction in the effect of AA in the colon. Sulfate-reducing bacteria are also involved in the etiology of UC, being the main producer of hydrogen sulfide in the intestine [50,51]. However, the effect of *Bacillus* spores or immunoglobulin on the amount of hydrogen sulfide in the gut or on lactic acid production has not yet been studied and could be a future research perspective.

The accumulation of ROS together with direct damage to epithelial cells has a negative impact on the release of proinflammatory cytokines, such as TNF-α or IL-1β, and thus increases inflammation [52]. In our study, MDA levels were increased in animals in the AA group compared to those in the control group. All agents tested in our study had a positive impact on MDA levels, significantly lowering the level relative to the AA group. MDA is a commonly used indicator of lipid peroxidation and oxidative stress [53]. Regarding TAC, the AA + MM group was the only one to demonstrate a significant increase relative to the AA group; all other groups exhibited a non-significant increase in TAC. These data are in accordance with data obtained in other studies and do not support the hypothesis that the production of free radicals in active colitis determines the total consumption of antioxidants [54]. Many studies have shown the effect of dietary amino acid supplementation on oxidative stress [55,56,57,58]. We speculate that MSB was able to restore the balance between ROS and antioxidants, as it has been reported that *Bacillus* spp. produce exopolysaccharide (ESP), which has significant antioxidant and immunoregulatory activities [38]. Furthermore, *Bacillus* spp. are able to maintain the levels of *F. prausnitzii*, which has anti-inflammatory and gut-barrier function protection properties, thus ameliorating UC symptomatology [59].

The severity of cellular damage induced by free radicals is directly related to the efficiency of the intracellular protection system; lipid peroxidation and the formation of hydroxyl radicals are prevented by antioxidant enzymes, including catalase [60]. The catalase activity reported in our study is in accordance with data obtained by other researchers [61,62]. In our study, plasma catalase levels slightly increased in the AA group, most likely because catalase reduce AA-induced oxidative stress. In groups treated with MSB or MM, the catalase level was significantly lower than that of the AA group. One explanation could be the decreased sensitivity of erythrocytes to oxidative damage in these groups. Another explanation is related to the protective effect of MSB or MM on the colon before colitis was induced, possibly implying a low level of oxidative stress that made a compensatory increase in catalase unnecessary. However, in the MSB + MM and MP groups, the catalase level increased slightly compared to the AA group. Such a stimulating effect on catalase activity has been previously described for other probiotics that were evaluated using different experimental models of colitis [63]. Regarding catalase, the results obtained were unexpected. It is possible that only the combination of MSB and MM is able to induce the production of an active catalase that can provide better antioxidant activity.

No histopathological changes were observed in the colonic mucosa and submucosa of the rats in the healthy control group. Beneficial effects of both individual administration of MSB or MM and especially of combined administration (MSB + MM) were observed in the histopathological evaluation of the colon segments. We observed intense inflammatory infiltrates in the mucosa and submucosa, moderate levels of neutrophils in the lamina propria, infiltration of neutrophils in the epithelium of crypts in more than 50% of crypts, erosion, and distortion of glandular architecture with goblet cell depletion in the colons from rats in the AA group (Figure 10). The worst RHI scores (RHI > 20) were obtained from rats in the AA group. RHI scores were lower in rats that were administered MSB, MM, MSB + MM, or MP, indicating that these agents were able to preserve tissue architecture (increasing crypt density and restoring intestinal crypt architecture) and to decrease the number of destroyed goblet cells; the best effect was reported for rats treated with MSB + MM and was similar to the effect of MP (RHI < 9 for both groups).

Experimental studies have shown that the administration of the serum-derived Ig preparations decreases proinflammatory cytokines, increases anti-inflammatory cytokines, and decreases intestinal permeability [63]. In a recent review, Yang et al. highlighted the effect of dietary amino acids on both intestinal health and function in pigs. They noted research demonstrating that cysteine increased the synthesis of mucin and glutathione, that lysine had an impact on the abundance of the intestinal microbial community, and that threonine increased serum IgG and the production of humoral antibodies [22]. The beneficial effects of the agents evaluated in our study, which contain *Bacillus* spp. or Igs with amino acids, are in line with the findings of the abovementioned studies.

Our study showed that overall, the results obtained from the group treated with MSB + MM were comparable to those obtained from the MP group. MP inhibits proinflammatory proteins such as NF-kB and AP-1, causing downregulation of proinflammatory cytokine expression and upregulation of the expression of other cytokines that reduce the production of inflammatory mediators (e.g. TGF-β). Prophylactic treatment with probiotics, as well as the use of other mucosal protective agents, can represent a valid management option in reducing the need of IBD medication (corticosteroids, intestinal anti-inflammatory drugs, monoclonal antibodies) or in reducing their side effects. Therefore, there is a need to develop more effective therapies for the treatment of IBD.

## 5. Conclusions

We conducted this study with the aim of determining whether probiotic bacteria such as *Bacillus* spp. and their metabolites, alone or in combination with immunoglobulins and amino acids, could participate in the cross-talk between intestinal and other cell types, thus influencing the innate immune response of the gut to acute injury induced by AA exposure in a rat model of UC.

The data collected in this study provide good evidence of the benefits of the combination of MM and MSB in an experimental model of IBD; it relieves inflammation (TNF-α, IL-6), oxidative stress (MDA, TAC) and it modulates the expression of adhesion molecules (VCAM-1, ICAM-1). Our data, along with the histopathological benefits, suggest that these supplements can partially prevent the structural and functional changes observed in the colon during colitis development. The beneficial effect is close to that of methylprednisolone, a known glucocorticoid agent used in severe UC. These data highlight the important role of dietary supplements in the prevention and treatment of IBD.
